# Exposure to violence, adverse life events and the mental health of adolescent girls in Nairobi slums

**DOI:** 10.1186/s12905-022-01735-9

**Published:** 2022-05-10

**Authors:** Yohannes Dibaba Wado, Karen Austrian, Benta A. Abuya, Beth Kangwana, Nicole Maddox, Caroline W. Kabiru

**Affiliations:** 1 African Population and Health Research Center, APHRC Campus, Manga Close, Off Kirawa Road, Nairobi, Kenya; 2 Population Council Kenya, Nairobi, Kenya

**Keywords:** Depression, Adverse events, PHQ-9, Adolescents, Slum, Violence

## Abstract

**Introduction:**

Mental health problems rank among the leading causes of disability among young people globally. Young people growing up in urban slums are exposed to adverse childhood experiences, violence, and other adversities. There is limited research on how exposure to violence and adverse life events influence adolescents’ mental health in urban poor settings. This study examines the associations between exposure to violence, adverse life events and self-reported depression in the slums of Nairobi.

**Methods:**

This study draws on data collected from 2106 adolescent girls aged 12–19 years who were interviewed in the third wave of the Adolescent Girls Initiative Kenya (AGI-K). Mental health was measured using the Patient Health Questionnaire (PHQ 9). Frequency distributions, bivariate chi-squared analysis and multi-variate regression models were computed to identify factors that are independently associated with depression.

**Results:**

About 13.3% of girls had symptoms of depression based on PHQ 9, 22% reported physical or sexual violence in the past year and about 47% of girls reported exposure to adverse life events in the family in the past year. After adjusting for the effects of socio-demographic factors, exposure to physical violence (AOR = 2.926, 95% CI 2.175–3.936), sexual violence (AOR = 2.519, 95% CI 1.637–3.875), perception of neighborhood safety (AOR = 1.533, 95% CI 1.159–2.028) and experience of adverse life events (AOR = 1.326, 95% CI 1.002–1.753) were significantly associated with self-reported depression. The presence of social support moderated the relationship between violence and mental health by reducing the strength of the association between violence and mental health in this setting.

**Conclusion:**

Given the magnitude of violence victimisation, adverse life events and depressive symptoms, there is a need to design interventions that reduce exposure to violence and provide psychosocial support to adolescents exposed to adverse events in urban slums in Nairobi.

## Background

Mental health problems, such as depression, rank among the leading causes of disability among adolescents and young people globally. According to the WHO, mental disorders account for about 16% of the global burden of disease and injury in people aged 10–19 years [1]. The WHO defines mental health as “the state of wellbeing in which the individual realizes his or her own abilities, can cope with the normal stressors of life, can work productively and fruitfully, and is also able to make a contribution to his or her community” [2]. Poor mental health can have detrimental effects on the wider health and development of adolescents and is negatively associated with several health and social outcomes such as alcohol abuse, tobacco use, illicit substances use, adolescent pregnancy, school dropout and delinquent behaviors [3, 4]. There is, however, very little research on the prevalence of depression and other mental disorders among adolescents in low and middle-income countries (LMICs). Furthermore, while there is evidence showing that young people growing up in the slums are exposed to adverse childhood experiences, gender-based violence, and other adversities, there is limited interrogation of how exposure to violence and other adverse life events in these settings affect adolescents’ mental health [5, 6].

Evidence from Demographic and Health Surveys (DHS) shows that violence against adolescents and young women is common in LMICs, with an estimated 28% of adolescents (ages 15–19 years) reporting experience of lifetime physical or sexual violence [7]. According to the 2014 Kenya DHS, about 35% of adolescent girls aged 15–19 years reported that they had experienced physical and or sexual violence since age 15 [8]. The few studies on adolescents in low-income contexts and slum settings in LMICs also show that violence against adolescents is widespread. For example, Hiremath and Debaje [9] found that 38% of adolescent girls in the urban slums of Mumbai, India had experienced domestic violence. In a study of school children in poor neighborhoods in Cape Town, South Africa, nearly 84% of school children reported exposure to some form of violence in their lifetime [10]. Previous studies from Nairobi slums also found that violence against adolescents and young women is common in the slums [11, 12].

Violence against women and girls has far reaching lifelong direct and indirect effects on the health, well-being and developmental trajectories of adolescent victims including injuries, unintended pregnancies, and increased risk of communicable disease such as sexually transmitted infections including HIV/AIDS [13–16]. Studies have also documented the mental health effects of violence and abuse [17–20]. However, there are relatively few studies that examine the association between exposure to violence and adolescents’ mental health. In a recent study of adolescent girls and young women in Kenya and Malawi, Mathur and colleagues (2018) found that violence perpetrated by non-partners was associated with increased levels of anxiety and depression [14].

Adverse childhood experiences (ACEs) such as violence and victimization, low peer, family or school connectedness, neighborhood safety problems and socio-economic problems are recognized risks to mental health [21, 22]. There is a growing literature that looks at the effects of adverse life events and neighborhood effects on health including in slum settings [23–25]. However, few studies examine the effects of neighborhood characteristics and exposure to adverse events on mental health of adolescents. Adolescents growing up in slum settings experience various adverse life events, including exposure to crime and violence, extreme poverty, poor housing, alcohol and drug abuse and delinquency [26]. As a result, adolescents living in slum settings may be at greater risk of mental disorders due to their living conditions, stigma, discrimination or lack of access to quality support and services [21, 22]. However, while exposure to violence, ACEs and perceptions of neighborhood safety are overlapping domains, we measured the three constructs separately in this study. Some studies from high income countries (HICs) defined ACEs broadly to include physical, sexual or emotional abuse, loss of parent, family discord and divorce, exposure to substance abuse and mental illness in the home, and violence in the home or neighborhood [27–29].

In situations of adversity and other risk factors of poor mental health, social support may play an important buffering role. Social support is a form of social capital that individuals can draw upon to help them cope with daily stressors [30, 31]. Several studies have documented the inverse relationship between social support and depressive symptoms among adolescents suggesting that social support may help protect adolescents against the negative effects of stressors and promote more positive mental health outcome [30–32]. In this study, we examine the extent to which exposure to gender based violence and adverse life events are associated with self-reported depression among adolescent girls in the Kibera slum of Nairobi and whether the relationship is moderated by social support.

## Methods

This study draws on data collected from adolescent girls aged 12–19 who were interviewed in the third wave of the Adolescent Girls Initiative Kenya (AGI-K)—a randomized controlled trial implemented in two marginalized communities in Kenya—Kibera slum in Nairobi and rural Wajir County. The AGI-K delivered multi-sectoral interventions (violence prevention, education, health, and wealth creation) to over 6000 girls ages 11–15 randomized into four intervention groups for 2 years. Details on the AGI-K study design and intervention arms are reported elsewhere [33, 34].

All survey rounds collected data on girls’ socio-demographic characteristics, schooling and educational attainment, social networks and self-efficacy, neighborhood safety, reproductive health knowledge, sexual behavior and HIV/AIDS in addition to the intervention components. The endline survey also included assessment of depressive symptoms and experience of physical and sexual violence in the past year before the survey. This study used endline data from Kibera slum in Nairobi to examine the association between gender-based violence, exposure to adverse events and depression among girls in the Kibera slum of Nairobi. Kibera is the largest slum in Nairobi, the capital of Kenya.

Depression was measured using the Patient Health Questionnaire (PHQ 9), a self-reported diagnostic measure of depression widely used and well validated in different settings and population groups including with adolescents [35, 36]. The PHQ 9 showed good internal consistency, with alpha level of 0.87. Each of the nine items were scored on a scale of 0–3, allowing a total score ranging from 0 to 27. The sum score (range 0 to 27) indicates the degree of depression, with scores of 5–9, 10–14, 15–19, and ≥ 20 representing mild, moderate, moderately severe, and severe levels of depression [37]. As the number of respondents reporting severe symptoms was very small, we combined all those with mild, moderately severe and severe depression to one category. Accordingly, respondents were classified as having symptoms of depression if they scored 5 and above on the PHQ-9 scale.

The main explanatory variables of interest were exposure to any form of violence (physical or sexual), perceived neighborhood safety, and exposure to household shocks/ adverse events in the past year. We used validated and standard tools to measure each of these constructs. Violence was measured using a shortened, modified version of the Conflict Tactics Scale [38] as used in DHS [39]. It asks about different acts of physical violence—having been pushed, shaken, or had something thrown at, slapped, punched with a fist or something that could hurt, kicked, dragged or beaten up, choked or burned on purpose—experienced in the last one year.

Sexual violence was assessed using the following items: physically forced to have unwanted sex or forced into other unwanted sexual acts or forced with threats or any other thing to perform unwanted sexual acts. An affirmative answer to one or more of items listed in the physical and sexual violence questions constitute evidence of physical and sexual violence. We focused on current prevalence of violence, defined as the proportion of adolescents who reported having experienced at least one acts of violence in the last twelve months.

Exposure to adverse events and perceptions of neighborhood safety were measured using survey questions adapted from the Global Early Adolescent Study (GEAS) [40]. To assess perceptions of neighborhood safety, respondents were asked whether they feel safe walking in their neighborhood in the day and in the dark; whether they have ever been robbed; whether they feel scared that they would be raped; or whether they have ever been touched indecently by a boy or a man in their neighborhood. Response options were coded as 1 for ‘yes’ and 0 for ‘no’ and a composite index of neighborhood safety was constructed from 6 questions which was recoded as ‘safe ‘ if the respondent did not endorse any of the neighborhood safety items and ‘not safe’ otherwise. Exposure to adverse life events in the past year was assessed with the following questions: whether anyone from the household died; whether household home was lost due to flood, fire or demolition; whether anyone in the household experienced major illness; whether a household breadwinner lost their job. Response options were coded as 1 for ‘yes’ and 0 for ‘no’ and was counted to categorize respondents households into those that experienced at least one event and those that had experienced none. For household food security, respondents were asked whether there was a day in the past month where the household went without food because there was not enough food in the household. Response options were coded as 1 for ‘yes’ and 0 for ‘no’.

Social support was measured using questions on parental connectedness and availability of support from close friends and adult members of society. The parental connectedness question asked adolescents ‘Do you feel close to your mother or female guardian?’ and ‘Do you feel close to your father or male guardian’? Response options were very close, somewhat close, not very close, not at all close and not applicable or don’t have one. Moreover, adolescents were asked whether they have a female friend in the community who they can turn to if they had a serious problem or with whom they can discuss their problems, joys and ask questions. Similar question asked whether they have a female adult in the community, other than their parents or teacher, who they can turn to if they had a serious problem or with whom they can discuss their problems, joys, and ask questions. Response options were ‘agree’ if they have and ‘disagree’ if they didn’t have one.

Data on socio-demographic variables (age, education, ethnicity, and religion, living arrangement), schooling and adolescent’s self-efficacy were also collected. Self-efficacy was measured using the self-efficacy scale adopted from the Global Early Adolescents Study (www.geastudy.com) that had been piloted in the slums of Nairobi [41]. Respondents were read each of the statements and asked whether they agree or disagree with each statement. Response options were coded as 1 for ‘agree’ and 0 for ‘disagree’. Participants were categorized into the three groups after transforming raw scores into z-scores where by participants with z-score of 1and higher were classified as having ‘high self-efficacy’; participants with z-scores ranging between -1 to 1 as ‘moderate self-efficacy’, and those scoring below − 1 as having ‘low self-efficacy’. The use of alcohol in the month before the survey and ever use of drugs/substance were not included in the analyses because these behaviors were only reported by a small proportion (about 1.1% and 1.3% respectively) of adolescents.

Data were analyzed using STATA Version 14. Frequency distributions were computed to describe adolescents’ characteristics. Bivariate chi-square tests were used to compare depressive symptoms by girls’ characteristics and multivariable regression models employed to identify factors that were independently and significantly associated with depression. Variables were selected for the multivariate analysis in two ways; based on evidence from previously published studies and strength of associations with the outcome of interest at the bivariate level (P-values < 0.05). All cases with missing values for one or more values of the key variables were excluded from the analysis. We examined the adjusted R-Square statistic and the F-test to select the most parsimonious model with estimates of adjusted odds ratio.

## Results

A total of 2106 adolescents were successfully interviewed at endline. Over two-thirds (73.6%) were aged 15–17 years while 18% were older than 18 years. Nearly 80% of the respondents were attending school at the time of the survey and about 84% of the adolescents attending school were at secondary level. Of those attending school, about 42% of them were in boarding school at the time of the survey. A small proportion of the adolescents (13.7%) reported engaging in any type of work for pay in the 12 months before the survey. Only about 3% of girls were previously (divorced or separated) or currently in marital union (Table 1).

**Table 1:** Percentage distribution of adolescent girls by exposure to violence, perception of neighborhood safety and adverse life events, Kibera

Variables	Physical or sexual violence	Neighborhood not safe	Any adverse event in the past year	Overall	
	(%)	(%)	(%)	N	%
Age of the respondent					
12–14	16.3	67.4	44.9	178	8.5
15–17	22.5	67.2	47.3	1549	73.6
18–19	23.7	58.5	44.7	472	17.9
Current school attendance					
Yes	21.7	66.7	45.9	1,678	79.7
No	24.3	61.8	49.3	428	20.3
Highest level of education					
No formal education	27.3	72.7	45.5	11	0.5
Primary	18.5	69.1	48.4	314	15.4
Secondary	22.6	65.3	45.8	1716	84.1
Have worked to earn income in the past year					
Yes	33.2	54.7	54.7	289	13.7
No	20.5	67.7	45.3	1817	86.3
Marital status					
Married/cohabiting	38.9	63.9	38.9	36	1.7
Separated/divorced	36.0	48.0	48.0	25	1.2
Never married	21.4	66.1	46.7	2018	97.1
Living arrangement					
With parents/caretakers at home	24.4	65.6	47.5	749	35.6
Boarding at school	19.1	67.8	44.8	886	42.1
Elsewhere (with relatives, alone, missing)	24.6	62.0	48.4	471	22.3
Respondent’s religion					
Catholic	20.0	67.1	47.1	490	23.3
Protestant	23.5	65.3	47.6	1,365	64.8
Muslim and other	17.9	67.0	37.3	212	10.1
Other (traditional or no religion)	28.2	56.4	56.4	39	1.9
Depressive symptoms					
None	18.3	67.9	45.8	1826	86.7
Mild	46.3	51.2	50.8	244	11.6
Moderate	60.7	50.0	57.1	28	1.3
Severe	50.0	62.5	62.5	8	0.4
Total (%, n)	22.2 (468)	65.7 (1384)	46.6 (981)	2106	100

About 22.2% of the adolescents reported that they had experienced physical or sexual violence in the year before the survey; 19.5% reported some form of physical violence while 6.4% reported sexual violence in the year before the survey. A higher proportion of adolescents aged 18–20 (23.7%), adolescents not attending school (24.3%), adolescents with no formal education (27.3%), those who worked for pay (33.2%) and married or cohabiting adolescents (38.9%) reported physical or sexual violence in the past year compared to their counterparts. The proportion of adolescents reporting any form of violence also varied by religion.

While 46.6% of adolescents reported exposure to any adverse life events in the past year, the proportion did not vary much by respondents socio-demographic characteristics (Table 1). The majority of adolescents (65.7%) felt that their neighborhood was not safe with considerable variation by socio-demographic characteristics. A greater proportion of younger (12–14 years), school going adolescents, adolescents with lower education, those living with relatives or alone as opposed to those living with their parents, those who worked for pay, and adolescents who were separated or divorced from their partners reported their neighborhood as unsafe. The overall proportion of respondents with combined mild, moderate and severe symptoms of depression on the PHQ scale was 13.3% (11.59 mild, 1.33 moderate and 0.37 severe depression respectively) (Table 1).

A greater proportion of older adolescents aged 18–20 years (15.7%) than younger adolescents aged 15–17 years (13%) or 12–14 years (10.7%) reported depressive symptoms (Table 2). However, the differences were not statistically significant. Likewise, adolescents with secondary education reported higher level of depressive symptoms (14.2%) compared with those with primary level of education (7.3%) and the difference was statistically significant. The proportion of adolescents reporting depressive symptoms was higher among those who worked for pay in the past year (21.3%), rated the safety of their neighborhood as unsafe (18.8%), were not close to their mother (22.4%), had a female friend who can provide help or advice when needed (14.0%), experienced any form of violence (physical as well as sexual) in the past year (28.6%), experienced adverse event in the past year (14.8%), and came from households with food insecurity (15.6%) (Table 2).

**Table 2:** Percentage of adolescents reporting depressive symptoms by socio-demographic characteristics and other risk factors, Kibera, Nairobi

Variables	No depressive symptoms		Depressive symptoms		Total	P-Value
	n	%	n	%	N	
Age of the respondent*						0.0221
12–14	159	89.3	19	10.7	178	
15–17	1347	87.0	202	13.0	1549	
18–19	317	843	59	15.7	376	
Current school attendance						0.270
Yes	1448	86.3	230	13.7	1678	
No	378	88.3	50	11.7	428	
Highest level of education						
Primary	291	92.7	23	7.3	314	0.003
Secondary	1471	85.7	245	14.3	1716	
None or missing education	64	84.2	12	15.6	76	
Living arrangement						
Living with parents	640	85.5	109	14.5	749	0.168
Board at school	766	86.5	120	13.5	886	
Living elsewhere	420	89.2	51	10.8	471	
Worked in the past year						
Yes	107	78.7	29	21.3	136	0.004
No	1719	87.3	251	12.7	1970	
Neighborhood safety						
Safe	1240	89.6	144	10.4	1384	0.001
Not safe	586	81.2	136	18.8	722	
Self-efficacy						
Low	438	88.3	58	11.7	496	0.079
Medium	951	87.3	138	12.7	1089	
High	437	83.9	84	16.2	521	
Parental connectedness (mother)						
Very close	1446	89.0	179	11.0	1625	0.001
Somewhat close	210	80.2	52	19.8	262	
Not close/no mother	170	77.6	4f9	22.4	219	
Parental connectedness (father)						
Very close	411	85.6	69	14.4	480	0. 341
Somewhat close	399	88.5	52	11.5	451	
Not close/no parent	1106	86.5	159	13.5	1175	
Has female friend who can provide help or advice when needed						
Yes	1509	86.0	245	14.0	1754	0.042
No	317	90.1	35	9.9	352	
Has female adult who can provide help or advice when needed						
Yes	1259	86.1	204	13.9	1463	0.186
No	567	88.2	76	11.8	643	
Experience of any form of violence in the past year(physical or sexual)						
No	1492	91.1	146	8.9	1638	0.000
Yes	334	71.4	134	28.6	468	
Adverse event in the in past year						
None	990	88.0	135	12.0	1125	0.046
One or more	836	83.6	145	14.8	981	
Household food insecurity						
Food secure	957	87.8	128	12.2	1085	0.023
Food insecure	869	84.4	152	15.6	1021	
Project intervention arms						
Violence prevention only	414	85.5	70	14.5	484	0.217
Violence prevention + Education	469	88.8	59	11.2	528	
Violence prevention + Education + Health	468	87.6	66	12.4	534	
Violence prevention + Education + Health + Wealth	452	85.0	80	15.0	532	
Total	1826	86.2	280	13.3	2106	

*Age missing for 3 respondents

Results of the regression analysis are presented in Table 3. Model I shows the unadjusted regression results. Few variables were significantly associated with self-reported depression in the bivariate chi-squared analysis as well as the bivariate regression analysis. Current school attendance, living arrangement, self-efficacy, paternal connectedness and project intervention arms were not significant in the bivariate chi-square analysis and were subsequently excluded from the multivariate regression analysis. After adjusting for the effects of age, education and other socio-demographic factors, exposure to physical, and sexual violence, perception of neighborhood safety, and experience of adverse life events in the household in the last year were significantly associated with self-reported depression in the model (Model II, Table 3).

**Table 3:** Odds ratio from regression analysis of the association of depression with violence, adverse life events and other risk factors (n = 2106)

Variables	Model I Unadjusted OR (95% CI)	Model II Violence and adverse life events AOR (95% CI)	Model III Full model AOR (95% CI)
Respondents age (RC = 11–14)			
15–17	1.280 (0.821–1.996)	0.900(0.531–1.525)	0.870 (0.512–1.480)
18–19	1.796 (1.106–2.917)*	0.956(0.525–1.738)	0.941 (0.515–1.719)
Highest level of education (RC = primary or lower)			
Secondary	2.098(1.441–3.054)**	2.055 (1.281–3.297)**	1.989 (1.232–3.211)*
Neighborhood safety (RC = safe)			
Not safe	1.881 (1.499–2.360)**	1.642 (1.258–2.143)***	1.533 (1.159–2.028)*
Experience of physical violence			
Yes	3.940 (3.102–5.006)**	3.141 (2.283–4.322)***	2.926 (2.175–3.936)***
Experience of sexual violence			
Yes	4.612 (3.310–6.426)***	3.148 (1.996–4.963)***	2.519 (1.637–3.875)***
Household food Insecure			
Yes	1.332 (1.063–1.668)*	1.259 (0.929–1.707)	1.217 (0.919–1.612)
Worked for pay in the past year			
No	0.532 (0.366–0.774)**	0.740(0.466–1.177)	0.835 (0.498–1.400)
Adverse event in the in past year			
Yes	1.275 (1.018–1.596)**	1.266 (1.113–1.762)*	1.326(1.002–1.753)*
Parental (maternal) connectedness (RC = very close)			
Somewhat close	1.892 (1.384–2.586)**		1.853 (1.280–2.683)***
Not close	2.564 (1.843–3.567)**		2.343 (1.552–3.538)***
Has close friends who can help or counsel upon need			
Yes	0.386 (0.011–0.760)*		1.304 (0.870–1.955)

*AOR* adjusted odds ratio

***p < 0.001; **p < 0.01; *p < 0.05

Model III controls for the effects of social support variables (parental connectedness and presence of good friends who can provide support) to examine the extent to which social support moderates the relationship between violence and self-reported depression. Although the strength of the association was reduced after adjusting for the effects of social support variables, both physical and sexual violence, perception of neighborhood safety and experience of adverse events in the past year all remained significant in model III. The odds of reporting depression were 53% higher among adolescents who perceived their neighborhood as unsafe (AOR = 1.533, 95% CI 1.159–2.028). The odds of reporting depression were three times as high among adolescents who were exposed to physical violence in the past year compared to adolescents not exposed to any form of physical violence (AOR = 2.926, 95% CI 2.175–3.936). Similarly, the odds of reporting depression were nearly two and half times as high among adolescents who were exposed to sexual violence in the past year compared to adolescent girls not exposed to sexual violence (AOR = 2.519, 95% CI 1.637–3.875). Exposure to adverse events in the family in the past year also increased the odds of reporting depression by about 33% (AOR = 1.326, 95% CI 1.002–1.753).

The social support variables also remained significant in the full model**.** With regards to adolescent’s connectedness to their parents, the odds of reporting depression were higher among adolescents not close (AOR = 1.993, 95% CI 1.336–2.975) or not very close (AOR = 2.606, 95%CI 1.658–3.098) to their mothers compared to those who were very close to their parents. The other variable on social support, availability of help and advice from close friends was not significantly associated with self-reported depression. (Table 3).

## Discussion

We examined the associations between exposure to violence, adverse life events and self-reported depression among adolescent girls in the slums of Nairobi. We found that violence victimisation and adverse life events are common among adolescents in the slums of Nairobi. Nearly one in four adolescents reported experiencing any form of violence (physical or sexual violence) in the past year before the survey. We acknowledge that our measure of adverse life events may be correlated with violence victimisation.

Evidence from the DHS also shows that about 28% of adolescent girls age 15–19 years in LMICs and one in four adolescents in sub-Saharan Africa experience some form of intimate partner violence [5, 42]. The few studies that focus on adolescents in slum settings in LMICs also show that violence against adolescents is common [6, 10]. Moreover, over two-fifth of adolescents reported adverse life events in the past year before the survey. Adverse life events experienced in the past year include death of household members, loss of home due to fire, demolition or natural disaster, major illness of members of the household and loss of job by a household breadwinner. Several other studies documented that such adverse life events are common in the slums of Nairobi [26].

Depressive symptoms were common with about 13% of adolescents reporting depressive symptoms in the two weeks before the survey. The prevalence of depressive symptoms falls within the range of prevalence estimates (10–20%) reported by the WHO [43]. A higher proportion of adolescents attending secondary education, adolescents who perceived their neighborhood as unsafe, adolescents with poor connection with their mothers and adolescents who experienced physical and or sexual violence as well as other adverse events in the household in the year before the survey reported depressive symptoms. After adjusting for the effects of socio-demographic characteristics, experience of physical and sexual violence in the past year, perception that the neighborhood is not safe and experience of adverse event in the family in the past year were significantly associated with depressive symptoms.

The effects of violence on the physical health of women and girls is relatively well documented [13, 15, 44] although few studies examine the association between violence and the mental health of children and adolescents in slum settings. These studies show that adolescents who experience violence are at greater risk for poor mental health than those who have not been victims of violence [14, 17–19]. We also found that the odds of reporting depressive symptoms is significantly higher among adolescents who experienced physical and or sexual violence, even after controlling for social support variables. Social support moderated the relationship between violence and mental health by reducing the strength of the association between violence and mental health in this setting. Strong social support play an important buffering role, protecting adolescents against the negative effects of stressors and promoting more positive mental health outcome [30, 32].

While exposure to physical and or sexual violence is a risk factor for mental health problems, evidence from other studies highlight the interlinkages between poverty and violence in influencing mental health. Poverty is a risk factor for both violence and mental ill health [10, 45] and the risk of violence is pronounced in low-income settings such as slums. Poverty also increases the risk of mental ill health in several ways; increasing the likelihood of mental health effects related to violence and adverse life events and also providing less of a buffer to protect against the consequences of those events [45]. Moreover, exposure to violence, adverse childhood experiences and perceptions of neighborhood safety are overlapping domains, as ACE definition and scores include exposure to violence as well as to high levels of crime in the neighborhood [27, 28].

Sexual violence occurs in the form of rape and forced sex, which happens to be shameful and stigmatizing to the victims increasing the risk of mental health conditions [14]. One study of sexual harassment and violence among high schools students in urban slums in Nairobi found that girls experienced frequent sexual harassment and violence in and out of school [46]. However, it is shown that the relationship between violence and abuse and mental health can be bi-directional [18, 19]. For instance, a systematic review and meta-analysis of longitudinal studies found association between depression and subsequent domestic violence and abuse. Domestic violence and abuse increases the likelihood of depression in women with no previous history of symptoms [18]. Many other studies indicated that increased exposure to violence increases emotional disorder in adolescence unless buffered by strong social support [10, 18, 47]. In addition, there are factors that may be associated with both exposure to violence and mental health. For instance, variables like age, education and neighborhood safety factors may predict both violence exposure and mental health.

Adverse life events significantly increased the odds of reporting depressive symptoms among adolescents. These adverse life events and the slum living conditions may predispose children and young people to stress and stress may lead to mental health disorders. Several studies including a systematic review of prospective studies have documented an association between adverse life events and mental health symptoms [48–50]. Regardless of the type of stressors, research has consistently shown that adverse life events predict subsequent risk of developing a major depressive episode [49, 51].

More than two-thirds of the adolescents reported that their neighborhood is not safe and such perception was significantly associated with depressive symptoms. While slums are characterized by poor and temporary housing conditions, they are often located in undesirable parts of the city, such as steep hillsides, river banks or industrial areas that may be unsafe [52]. Studies also show that crime is more prevalent and severe in urban slums [53]. Moreover, there is a large body of literature that looks at neighborhood effects on health including in slum settings [23, 25]. Research shows that people who live in slums share environmental risks, and injury from accidents and violence, which tend to be among the major causes of death in slums [54, 55]. One systematic review that assessed the health vulnerabilities of children in slums found that children living in slums have more behavioral and emotional problems than children living in non-slum urban areas or rural areas [56].

There was no significant difference in depressive symptoms between the various arms of the intervention. As the AGI-K project did not include any specific intervention focused on adolescent mental health or psychosocial wellbeing, the intervention may not have had an effect on girls’ mental health. Further, the project included a community level intervention (community engagement) focused on violence prevention provided to all study arms.

The study is not without limitations. As a cross-sectional study, the data is affected by recall and reporting bias. First, violence victimization may be under reported given the culture of silence that surrounds violence. Similarly, mental health may be under reported due to the stigma associated with mental illness. Data on mental health were collected at endline only. As such, the design of the study also precludes causal inferences. The analysis did not look at differences by severity of the depression because the number of respondents with the severe depression is very small limiting the statistical power to disaggregate the analysis. The analysis on violence focused on physical and or sexual violence and did not include emotional violence. Yet emotional violence is an important component of violence perhaps with significant effects on mental health. In addition, as data used for this study were collected after interventions addressing violence prevention, education, health and wealth creation, exposure to the interventions may have biased the results.

## Conclusion

The study showed that depression is more common among adolescents in the slums who experienced sexual violence, physical violence, and adverse life events and perceived that their neighborhood is unsafe. The slum environment is characterized by dire poverty, insecurity and violence, which increase the risk of mental health among adolescent and young people. Thus, interventions that reduce exposure to gender based violence and provide psychosocial support to children of families exposed to adverse events are required in marginalized urban setting such as the Kibera slum. More importantly, there are effective strategies to ending violence against children and adolescents such as the WHO’s INSPIRE strategy, that can be adapted and implemented to prevent and respond to violence against children and adolescents.

## Data Availability

The data used for this manuscript can be made available upon request from the corresponding author.

## Abbreviations

AGI-K: Adolescent Girls Initiative Kenya
AIDS: Acquired immunodeficiency syndrome
DHS: Demographic and Health Surveys
GBV: Gender-based violence
HIV: Human immunodeficiency virus
PHQ: Patient Health Questionnaire
WHO: World Health Organization
